# Screening and identification of novel biologically active natural compounds

**DOI:** 10.12688/f1000research.11221.1

**Published:** 2017-06-05

**Authors:** David Newman

**Affiliations:** 1 Newman Consulting LLC, Wayne, PA, USA

**Keywords:** natural compounds, natural products, screening, bioactive materials

## Abstract

With the advent of very rapid and cheap genome analyses and the linkage of these plus microbial metabolomics to potential compound structures came the realization that there was an immense sea of novel agents to be mined and tested. In addition, it is now recognized that there is significant microbial involvement in many natural products isolated from “nominally non-microbial sources”.

This short review covers the current screening methods that have evolved and one might even be tempted to say “devolved” in light of the realization that target-based screens had problems when the products entered clinical testing, with off-target effects being the major ones. Modern systems include, but are not limited to, screening in cell lines utilizing very modern techniques (a high content screen) that are designed to show interactions within cells when treated with an “agent”. The underlying principle(s) used in such systems dated back to unpublished attempts in the very early 1980s by the pharmaceutical industry to show toxic interactions within animal cells by using automated light microscopy. Though somewhat successful, the technology was not adequate for any significant commercialization. Somewhat later, mammalian cell lines that were “genetically modified” to alter signal transduction cascades, either up or down, and frequently linked to luciferase readouts, were then employed in a 96-well format. In the case of microbes, specific resistance parameters were induced in isogenic cell lines from approximately the mid-1970s. In the latter two cases, comparisons against parent and sibling cell lines were used in order that a rapid determination of potential natural product “hits” could be made. Obviously, all of these assay systems could also be, and were, used for synthetic molecules.

These methods and their results have led to a change in what the term “screening for bioactivity” means. In practice, versions of phenotypic screening are returning, but in a dramatically different scientific environment from the 1970s, as I hope to demonstrate in the short article that follows.

## Introduction

Over the last 40 or so years, bioactive natural product discovery and development has moved from what was known colloquially in the trade as “grind and find techniques”, also termed “determining the phytochemistry of the plant”, to the use of very advanced analytical, genomic, metabolomic, and informatic techniques together with high throughput and/or “high content screening”.

In the days when the author was active in an industrial laboratory as a bench scientist, mainly in antibiotic discovery (late 1960s through the early 1980s), the methods always involved the screening of compounds or extracts against microbial cells, usually a relatively safe surrogate, such as the standard FDA strain of *Staphylococcus aureus* known as “209P”, a common *Escherichia coli* strain, and a suitable *Candida albicans* strain, all designed to show activity that could then be followed in a bioactivity-driven isolation format. During this time frame, “crude pre-fractionation systems” were utilized that consisted of adsorption or desorption through different ion-exchange systems, macro-reticular resins (based on XADs), silica gel, and alumina columns, sometimes independently, other times in series. Since these columns were handmade using Pasteur pipettes (the “Sep-Pak systems” were well in the future) and high-performance liquid chromatography (HPLC) was unknown until the late 1970s, the throughput was slow but effective in determining some of the potential chemical classes of antibiotics, aminoglycosides for example, that were in the crude extracts. Semi-purified extracts from these processes would then be tested *in vivo* in suitable mouse models of infection. Similar techniques were also used by scientists (including the author) when investigating antitumor activities in crude extracts of marine invertebrates and an occasional plant extract. Then, a simple mouse leukemia (L1210 or P388), or at times KB or HeLa (confluent mammalian), cell line was used to follow biological activities, followed by *in vivo* studies in both situations in either syngeneic (usually at the time) or SCID or *NuNu* mice if the facilities were available.

In contrast, most of the investigators who were not connected to the then-large-scale program run by the US National Cancer Institute (mid-1960s to 1981), and who were using plants as a source, would conduct isolation and purification of compounds from a plant extract (determining the phytochemistry of the source) and then, once the compound had been identified, might have a proportion of the pure materials tested in any “available assay”, hence the use of the soubriquet “grind and find” for this process. Even today, this system is still in place, mainly for plant-derived materials, in countries where the scientific infrastructure is not conducive to cell-line and/or animal screening owing mainly to the cost of the infrastructure required to maintain such facilities.

To perhaps the chagrin of a considerable number of natural product chemists, and in particular botanists and marine biologists, it is now becoming evident that molecules that have very significant biological activities from a pharmaceutical perspective are often not produced by the organism from which they were isolated but are the product of either microbes that are “in, on, or around” the source organism, or are perhaps the product of “chemical talk between organisms”, with at times the nominal producer being only a “container” for the single-celled organism/organisms that is/are the source.

What I hope to demonstrate is how the interplay of genomics and metabolomics coupled to mass spectroscopy (MS ^n^) and informatics has led to what can be classified as a revolution in both screening and isolation, with novel screens (or not as will be seen later) coupled to the very rapid identification of compounds. In some respects, a very complex “grind and find” operation, in others a very sophisticated analytical screening, followed by the isolation of active entities using a multiplex approach.

The unifying principle might be, if I am allowed a little humor, “what you isolate may not be produced by what you thought was the source”, and I will show that, as a result, Mother Nature still has many tricks to show us.

## Screening secondary metabolites: nominally from all sources (microbe, marine invertebrate, and plant)

### Preamble

What I hope to do is, by utilizing the data from a series of published research papers and reviews from 2012 to date (together with some earlier papers that demonstrated the necessary science), show that the term “screening for bioactive agents” covers a number of related approaches, with the advances in the rapid identification of secondary metabolites over the same time period being due to one major method, that of MS ^n^, or multiplexed mass spectral approaches, with the use of HPLC or, in its absence, and as shown later, nuclear magnetic resonance (NMR) profiling of enriched fractions. The utilization of these techniques coupled to very rapid next-generation sequencing of gene clusters and/or total genome sequencing and significant advances in informatic analyses of the data obtained have caused a major paradigm shift in “the concept of screening”.

A major emphasis, as might be evident from the introductory comments, is on microbes, both currently cultivatable and those that are not yet able to be cultivated. The use of cultivatable microbes is obvious, but one may ask, how on earth can you utilize the as-yet-uncultivated organisms? I will show how this has been done successfully and the extremely interesting findings that have come from such efforts.

### Screening methods and perceived current practice

Anyone who reads the literature related to the discovery of bioactive materials, irrespective of whether the sources are natural products, modified natural products, or synthetic compounds, has realized that, over the last 25–30 years, the (perceived) paradigm changed from phenotypic screening (usually cell or animal based) to the use of isolated “targets”. The reasons for this change are quite simple but not often mentioned. The “collision” of the invention of the 96-well plate, the rise of recombinant DNA technologies, and access to cheap and simple computing platforms (the PC) permitted the rapid production of targets (enzymes or proteins), their “interrogation” by semi-automated to automated systems, and the analyses of results in a short time period.

This led to the realization that the numbers of available chemical compounds were much too low, and since the screens were run in campaign-mode, the time frame, usually less than 3 months for a given screen, was totally unsuitable for the screening of other than pure natural products in the late 1980s to mid-1990s. This lack of compounds to test led to the initial rise of combinatorial chemistry, which promised to solve the supply and any intellectual property problems, since the compounds produced were only in a particular laboratory or company. Literally millions of compounds (pure and semi-pure) were screened in massive numbers of screens, and compounds were identified as “hits or leads”, but miniscule numbers actually reached preclinical status and then clinical trials.

In practice, if one looks at the analyses of sources of drugs since 1981 to 2014, there are perhaps three approved drugs worldwide that are *de novo* combinatorial discoveries, with one being discovered by the use of fragment techniques on targets ^1^. Combinatorial chemistry is magnificent for the development of an existing lead, but not for *de novo* discovery. Further examples of the problems with drugs approved from the use of target-based screens (in the area of anticancer drugs) are their side effects. As Fabbro (the biologist behind Gleevec ^®^) explained in 2014 ^2^ and 2015 ^3^, people forgot (or did not realize) that multiple types of kinases are not only present in cancer cells but also essential components of all cell metabolism.

To add to the confusion surrounding “target choices”, a paper published in late March 2017 by a group from Cold Spring Harbor ^4^ throws doubt upon a choice of a particular kinase (MELK) in triple negative breast cancer. In this paper, MELK, the target of two drugs thought to be MELK inhibitors from kinase testing and currently in clinical trials, was shown not to be the target by use of CRISPR/Cas9 technology, even though the original target was “correlated with RNAi inhibition”. By knocking out the “MELK target” and still demonstrating excellent cell growth inhibition, the target of these clinical candidates is now open to question. Thus, in this particular case, target-based screening is “debatable”. How many more such “problems” are yet to be found using this technology is now an open question.

A very interesting recent review was the thorough analysis done of all drugs approved by the US FDA for the treatment of cancer between 1999 and 2013 by Moffat *et al.* ^5^ This review built on the earlier report by Swinney and Anthony, who in 2011 demonstrated that, of the 183 small molecule drugs approved across diseases between 1999 and 2008, 58 (32%) were from phenotypic screens, and if one considered new entities that were “first in class”, then 28 of the 50 were from phenotypic screens and 17 were from target-based systems ^6^.

Do the figures in the paragraph above mean that one should go back to phenotypic screening or continue with targeted methods? There is no one answer to this question, but what has been occurring over the last 7-plus years, because of the significant advances mentioned in the opening paragraph of the preamble, is that a very up-to-date version of “grind and find” has now effectively taken over the initial screening systems, at least in the case of microbial secondary metabolites.

Discussions centered around such organisms will be the major focus of the rest of this review for the relatively simple reasons that a large percentage of all marine invertebrate-sourced natural products are the result of the interplay between microbes and their hosts. In addition, it is now becoming evident that a similar relationship may occur with a significant number of plants, in particular with their fungal endophytes, and there are now many examples of insects using microbes as sources of defensive metabolites ^7^.

## Modern versions of “grind and find” and their use in screening

### Application of single-cell genome interrogation to natural product structures

More than 20 years ago, the biotech company Diversa built upon some earlier research by a smaller biotech company, One Cell, where environmental microbes were diluted out and effectively suspended in a single drop of medium. This system allowed the growth of some previously uncultivated microbes but was not further followed up once Diversa folded. Recently, a Japanese group published a method using water-in-oil droplets to ferment such organisms but either were unaware of the much earlier work or did not locate any relevant references to it ^8^. However, they did reference a 2014 paper in Nature ^9^ that demonstrated not the fermentation of as-yet-uncultivated microbes but the isolation of single cells and subsequent DNA amplification that permitted the identification of the biosynthetic pathways of what were thought to be, up to that time, marine-sponge-derived bioactive metabolites.

In 2014, the Piel group at the ETH in Zurich published the paper mentioned above, which effectively revolutionized the understanding of the source of over 30 bioactive marine sponge-sourced secondary metabolites ^9^. The sponge *Theonella swinhoei* Y (Y for “yellow variant”) was a well-known producer of bioactive compounds with over 30 bioactive structures determined over the years. These compounds came from a combination of classical isolation and then biological activity determination and the use of bioactivity-driven isolation depending upon the compound.

**Figure 1.**
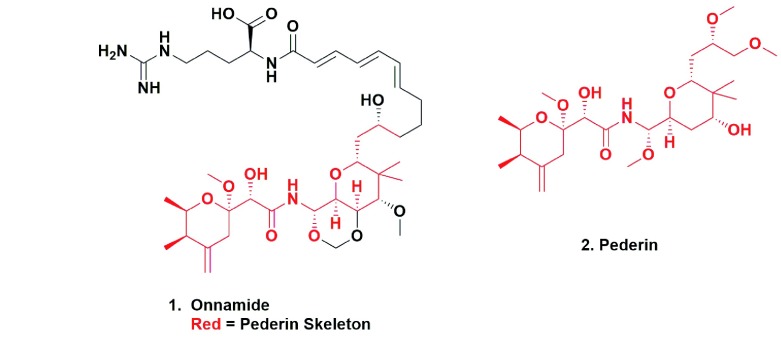
Onnamide and Pederin. Onnamide and Pederin.

**Figure 2.**
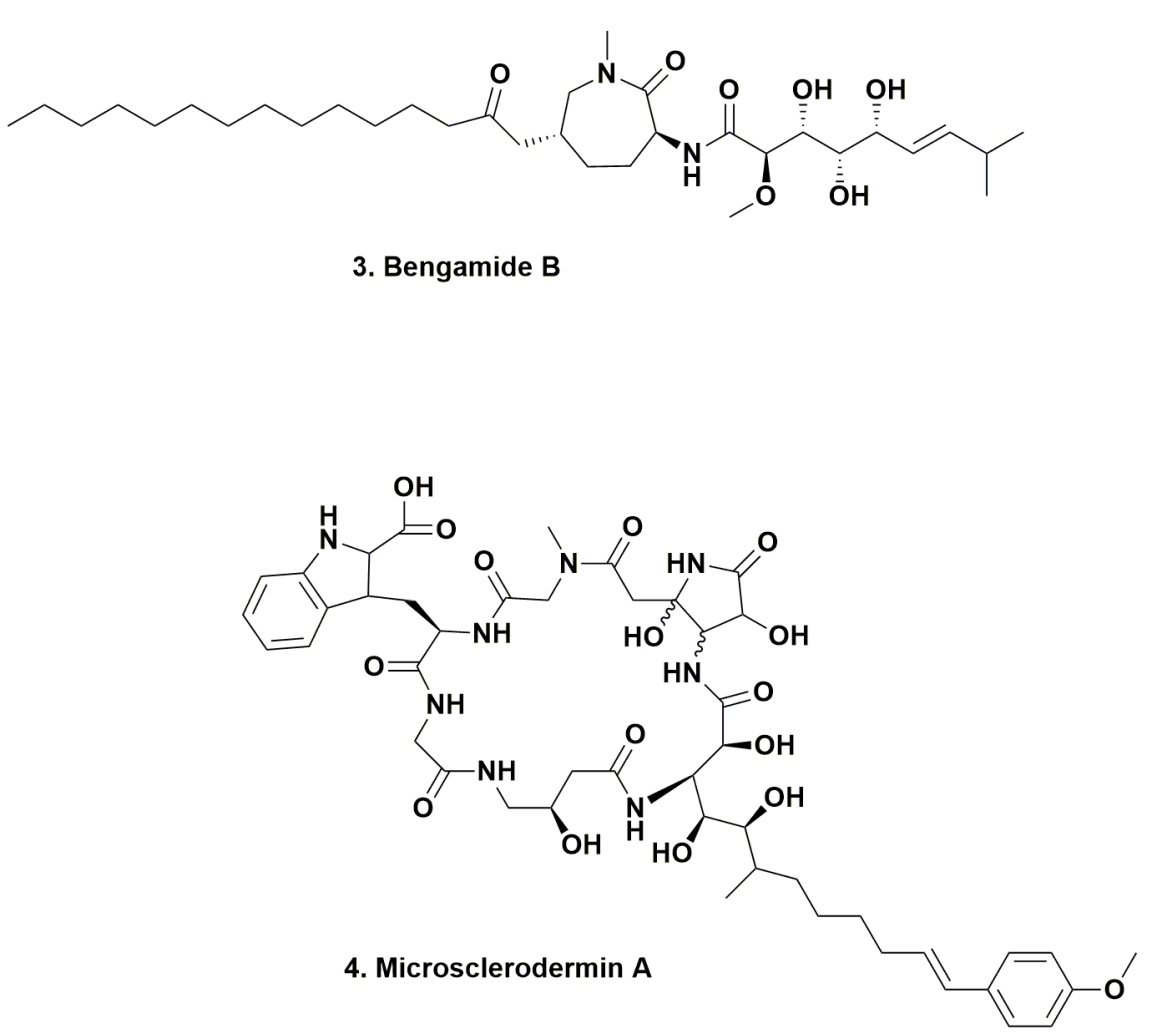
Bengamide B and Microsclerodermin A. Bengamide B and Microsclerodermin A.

In this paper, the group demonstrated, by the isolation of single bacterial cells from the whole sponge macerate, that the previously unknown and currently uncultivated microbe had the genomic potential to produce the widely disparate structures previously found, including the very potent agent onnamide ( Figure 1, [1]), a pederin-based ( Figure 1, [2]) molecule originally found in the Brazilian blister beetle. The story of the work performed over more than 30 years that led to the identification of pederin as a microbial product was covered in a recent publication ^7^. The techniques of DNA amplification from a single microbe through to the determination of biosynthetic methods are now being applied to other marine-derived secondary metabolites, and two recent papers from the Piel group should be consulted for current information ^10, 11^. However, it should be pointed out at this stage that this is only the “tip of the iceberg” as far as any production system is concerned. Although it might be feasible to use *Theonella* as a host in aquacultural production, as was done by the New Zealand group ^12^ in the 1990s to produce small quantities of halichondrin B, it will probably need to be transferred into a heterologous host in order to produce any metabolite from this discovery.

### Mass spectral *in situ* determination of secondary metabolites

In 2009, Esquenazi *et al.* published a review of the techniques that could visualize the production of secondary metabolites in microbes during their growth phase and, in particular, demonstrated the utility of imaging MS ^n^ (IMS) ^13^. This methodology permitted researchers to follow secondary metabolite production in bacterial cells by using the mass spectrum of the chosen metabolite as the sensor. In two later reviews in 2011, the Dorrestein group, of which Esquenazi was then a member, demonstrated that the technique was of significant import in determining the spatial production of metabolites and that one could also recognize the presence and production of novel metabolites ^14, 15^.

That this was not just a technique for demonstrating production at a single time point was shown in 2012, when Xu, working with other scientists at the Scripps Institute of Oceanography, used the technique to demonstrate the production of didemnin B ^16^. This compound, prior to a single report in 2011 ^17^, was thought to be the product of an encrusting ascidian and was the first directly marine-sourced compound to enter clinical trials as a potential antitumor agent. In the report from the Scripps group and their collaborators, the authors not only demonstrated the production of didemnin B but also, by growing the producing microbe on a suitable matrix that supported the use of IMS, were able to follow the time course of didemnin B production in the “fermentation” for the first time. The sequence of the production of “putative” intermediates leading to the final molecule, didemnin B, in the biosynthetic pathway was determined directly, thus proving the proposed biosynthetic pathway ^16^.

### Linkage of mass spectroscopy and genetic sequences as primary screening systems

With the examples described above as proof of the utility of the technique, a substantial number of papers have been published over the last 4 years or so showing the capabilities of MS ^n^ as a screening tool either coupled to genomic sequences or recognizing the production of potential novel compounds before biological screening. Although the advent of next-generation sequencing permitted the identification of biosynthetic gene clusters, either as part of a full sequence determination or as isolated entities, a major stumbling block was in proving that a given gene cluster produced a metabolite of interest, though significant numbers of linkages were obtained, usually by comparative studies.

Thus, by linking mass spectral analyses to genomic sequencing, investigators could link the production and identification of a given series of molecules to a particular microbe and then frequently identify the potential genomic cluster. Doroghazi *et al.* in 2014 ^18^ published the initial results of an analysis of 830 actinomycete genomes that included 344 new total genomes and 412 gene clusters that were listed in GeneBank and produced known secondary metabolites. Using a significant number of computational tools, the data obtained were grouped into 4,122 gene cluster families (denoted as GCFs) containing 11,422 gene clusters. The clustering or metabolite production was validated in a significant number of instances by mass spectral analyses of the metabolites produced. What was also of interest were the numbers of natural product gene clusters found per taxonomic family, with the well-known families having averages around the lower 20s per genome analyzed. Does this mean no duplication across families? No, but the technique permits a rapid deconvolution in due course, particularly when coupled to the results of other techniques that will be discussed below.

In 2016, Henke and Kelleher published an excellent short review on the utility of MS ^n^ techniques to dereplicate compounds from microbes using a “structure-based approach” rather than a bioactivity-driven approach ^19^. Effectively, MS ^n^ techniques came to the rescue of a “grind and find” process. In addition to this review, there were other significant reviews covering the use of these techniques that should also be consulted by interested readers. The limitation of length in this review means that discussion of these other papers covering the utility of this approach cannot be covered, but the following recent papers are well worth consulting ^20– 28^.

### Gene maps as leads to novel natural product bioactivities

There are two databases that have the potential to help in the screening of both fractionated extracts (see next section for a discussion of such libraries) and pure compounds. The largest is the “connectivity map”, also known as “cmap”, located at the Broad Institute of MIT and Harvard in Cambridge, Massachusetts ^29^. Quoting from their website at https://portals.broadinstitute.org/cmap/, the cmap “is a collection of genome-wide transcriptional expression data from cultured human cells treated with bioactive small molecules and simple pattern-matching algorithms that together enable the discovery of functional connections between drugs, genes and diseases through the transitory feature of common gene-expression changes”. Although there do not seem to be any published results from screening of natural product extract libraries, there definitely is significant potential for future use.

However, a smaller database specifically designed to link natural product isolation, in this case from marine-sourced microbes, with notation of a possible mechanism of action was constructed by MacMillan and White at the University of Texas, Southwestern Medical Center. A recent paper has demonstrated its success in working with marine-derived natural products and leading to the identification of a novel AKT inhibitor ^30^.

### Pre-fractionation of natural product libraries

What has occurred in the last few years is the realization by natural product chemists and biologists that extracts from any natural product source are not generally amenable to high-throughput screening, particularly against enzymes/receptors. Though there have been recent reports of successful programs using the crude microbial extracts from the NCI microbial extract libraries ^31– 33^, their costs were significant and were paid for by some version of a grant or contract.

There are also potential problems involved in dereplication in any system that utilizes the same or a similar microbe in multiple media and growth conditions, which were part of the sources of the NCI microbial extract collection, where microbes could have been fermented in up to 12 different media conditions commencing in the late 1980s. This multiple media approach has been entitled OSMAC (one strain/many compounds) and is often ascribed to work reported in 1999 by Schiewe and Zeek ^34^. This attribution is incorrect, although it has entered the literature since the process was first described from an academic aspect by Zahner in 1977 ^35^ and had been in general use in the antibiotic discovery programs in the pharmaceutical industry for at least 17 years prior to the Zahner paper. Since industry generally did not publish their techniques (the author was using such systems before 1970, and they had been in general use since the early 1960s), such information is not in the general literature, though the NCI contract conditions that led to their microbial collection were written by a retired microbiologist from Squibb and published in the mid-1980s specified just such an approach.

Thus, in order to reduce the problems associated with crude microbial extracts in particular, together with plant and marine-invertebrate extracts, scientists continued to think about how to optimize extracts for screening. The first formal publication from academia on the concept of pre-fractionation of crude extracts was probably the paper in 1999 by Schmid *et al.* from the Hans-Knoll Institute in Germany, quoting the use of Zymark SPE work stations for such fractionations ^36^. Though perhaps the first academic link, the concept had been used in various ways in small and large companies for years before then. Small companies in the early 1990s, such as Xenova in the UK, used pre-fractionation with HPLC tracing. Earlier, as mentioned above, in the late 1970s, larger companies such as SK&F (now GSK) used much cruder versions of the same concept owing to analytical limitations (personal observation by the author). From 2002 to 2008, companies such as Sequoia (specializing in plant-based materials from the Missouri Botanical Gardens) ^37^, bioLeads GMBH ^38^, and MerLion, specializing in microbial and marine invertebrate extracts ^39^, together with Wyeth ^40^ published their methodologies, with probably the first academic group to publish after the Hans-Knoll group mentioned earlier being the Ireland group at Utah in the later 2000s ^41^.

All of the fractionation methods used from the late 1990s were designed so that individual “wells or tubes” would in general contain only 3–10 compounds, and from the early 2000s analytical data on the pre-fractionated materials was obtained during the process ^41– 47^. It should be noted, however, that the initial impetus for fractionation before assay came from industry or from groups that started in industry and then moved into academia (i.e. ** Quinn at Astra Zeneca and then Griffith University, and Butler at MerLion and then University of Queensland).

Although all have overlap in their coverage, the following three review articles are worthwhile extra reading on the pre-fractionation topic. The 2013 review by Henrich and Beutler includes the pre-fractionation techniques used at the NCI that utilize the NCI’s Natural Products Repository ^44^. The 2014 chapter on Marine Bioprospecting by Fenner and Gerwick covers the usage of marine-derived extracts ^47^, whilst the 2015 review by Gaudencio and Pereira covers the whole period from 1993 to mid-2015 ^48^.

### Utility of fractionated natural product libraries and NMR profiling

Another instrumental technique that can be said to have “come of age” in screening processes is the use of NMR profiling of natural product extracts as a screening tool. The use of what could be termed “hyphenated-analytical systems including online NMR” in the initial profiling/screening of compounds as they eluted from (usually) HPLC systems has a relatively long history, with one of the first online NMR analyses of single compounds as a method of detection published 20 years ago ^49^.

The Eskitis group at Griffith University used their modification of pre-fractionation to analyze solid-state fermentation extracts of *Streptomyces* strains isolated from termite gut. These extracts were fractionated into lead-like enhanced (LLE) fractions using published methodology from their laboratories ^43^. This process led to a dataset of 420 LLE fractions and each was subjected to NMR profiling, with the spectra being manually examined for the occurrence of unique chemical profiles. They were classified as non-repetitive or unique NMR resonances and then followed further dereplication by linking specific spectral types to the previously collected distinctive ESIMS ion peaks (these were collected during the LLE process). These processes enabled the identification of six new secondary metabolites in addition to five known metabolites ^50^.

### Unbiased phenotypic screening with pure natural products in high content screens

Two excellent papers were published in 2016 from the Quinn group in Australia demonstrating how to couple phenotypic screening against non-immortalized human olfactory neurosphere-derived (hONS) cells (primary cells derived from Parkinson’s disease patients) with pure natural products using a high content screening system. The first screen covered isolated metabolites from marine sponges ( *Jaspis splendens*) to prove that the overall system could function ^51^. The following screen then utilized a 500-plus pure compound set from the Nature Bank collection at Griffith University ^52^. The results demonstrated that such a high content screen produced multiple possibilities for the identification of the interaction(s) with cellular organelles/protein interactions, being limited only by the specificity of the fluorescent probes used to demonstrate the responses.

To further demonstrate the potential of such high content screening, recently an excellent review covering almost all aspects of high content screening and its associated operations was published by a German group from the Helmholtz Centre in Braunschweig (the old GBF). This should be read in order to see the progression in techniques and technologies from 1997 to the end of 2015 ^53^.

### Metabolomics meets modern assay techniques

In a review paper in 2015 ^54^, Kurita and Linington covered the various techniques that now permit results from high content screening of extracts to link phenotype and chemotype and, later that same year, published an excellent paper on what they call “compound activity mapping”, which integrates high content biological screening and untargeted metabolomics to identify potential compounds with activity ^55^. This article should also be read in conjunction with the comments above on “cmap” and “FuSiOn” databases.

Although most scientists working with microbes have tended to work with eubacteria, predominately actinobacteria, there are two groups who have worked in other taxa for many years: one with fungi, predominately *Aspergillus* species, and the other with myxobacteria.

In the case of fungi, although it is not yet feasible to survey the complete secondary metabolome of a single fungus, there is the potential for 80–100 putative biogenetic clusters to be recognized from full genome studies in *Aspergillus* strains ^56^. In addition, the activation of cryptic clusters via epigenetic “tricks” also demonstrated further potential in these organisms ^57^. As a result, their potential is really only just being recognized ^21, 58– 60^.

In the case of the myxobacteria, which are predatory Gram-negative bacteria, just as in the case with the Keller group at the University of Wisconsin and *Aspergillus*, one research group, the Müller group in Germany (the lineal descendant of the Reichenbach and Höfle group at the then GBF in Braunschweig), are the pre-eminent investigators of secondary metabolites from this unusual taxon. In the last 4 years, they have published some excellent articles on this particular group of microbes, demonstrating their potential as sources of compounds with unusual structures and biological activities ^61– 64^. In addition, what is relevant to the comments earlier about actual sources not being what they were thought to be, in 2015, the Müller group plus a group from Sanofi in Germany reported not only the isolation of bengamides ( Figure 2 [3]; a series of compounds originally isolated from *Jaspis* sponges) from terrestrial myxobacteria but also the details of large-scale production and optimization of the base structure ^65^. The initial report of production of the bengamides from a terrestrial microbe was in fact in a patent application from Sanofi-Aventis in 2003, with the patent’s international filing date being in October 2004 ^66^. This demonstrates that the claim for the first report showing the material from a myxobacterial source from the Crews’ group in California in 2012 ^67^, who had reported the marine-sourced bengamides many years previously, is not correct and emphasizes that the patent literature can hold very significant information that is often not checked by academic scientists. This taxon also produces the microsclerodermins ( Figure 2, [4]), agents originally reported by Bewley *et al.* in 1994 from a lithistid sponge and then, just as in the case of the bengamides, reported by the Müller group in 2013 from a terrestrial myxobacteria ^68^.

### Statistical tools for the interrogation of results

Although the production of spectral data of all types is now a “relatively simple process” if one has the necessary resources, the conversion of such datasets into usable information, structures, linkage to targets etc. is a process that requires the use of multiple statistical tools, thus the processes are comparable to the use of “chemometrics” in chemical operations. Chemometrics is usually defined as “relating measurements on a chemical system or process to the state of the system via application of mathematical or statistical methods”, the use of principal component analysis (PCA) being just one example of the tools available.

In order to link information from multiple sources to “screen” natural products, a number of groups, some referred to earlier, have used “chemometric methods” linked to biological datasets so that initial findings from one analytical system can relatively easily be used to link to previously identified compounds, biological targets, genomic sequences, and metabolomic datasets across taxa. The datasets used are not limited to just natural products but can, and do, cross into information derived from synthetic or semi-synthetic compounds that have reported biological activities. Recent examples are the articles (some referred to earlier) from the Dorrestein group and collaborators ^22, 24, 69, 70^ and the Linington group and collaborators (also referred to earlier) ^55^. In addition to these, recent papers that deal with the chemometric link are one by Humbeck and Koch ^71^ and two papers from the Oberlies group ^72– 73^.

### Epigenetic modulation of source microbes

An area that may well have significant relevance in the future is (in addition to “persuading” as-yet-uncultivated microbes to grow, thus enabling the use of the vast array of techniques that can be used in fermentation processes) the activation of the so-called “cryptic clusters” in microbes. Just as in the human genome, the full epigenetic control mechanisms have not been elucidated in any microbe. Yes, we do know some of the parameters required in certain biosynthetic clusters, but how much of the total genome of a microbe is composed of control mechanisms is unknown.

There are relatively simple methods and more complex ones that can be used to “unlock” the Pandora’s box that any genome represents. The modification of genetic control elements in *Aspergillus*, as published by Bok *et al.* in 2006 ^57^, is one route, but the use of exogenous chemicals such as DNA methylators or histone acetylation modifiers are simpler examples, with some excellent work demonstrating such possibilities emanating from the Cichewicz group in 2008 ^74^ and expanded in following years ^75– 78^.

In addition to the “epigenetic modulators” listed above, the use of simple antibiotics in a single microbial fermentation can lead to the production of previously unknown metabolites. Thus, Truong *et al*. demonstrated that if *Burkholderia thailandensis* is treated with the well-known antibiotic trimethoprim, the activation of an orphan LuxR homolog known as MalR occurs, which is linked to the gene cluster that yields the toxic polyketide malleilactone ^79^. This sublethal trimethoprim– *B. thailandensis* combination was then later shown to induce the production of over 100 compounds previously not known from this particular microbe ^80^. In order to identify these materials, the investigators used the mass spectral networking technique pioneered by the Dorrestein group ^70, 81^. A very recent paper from the same group then identified the master regulator Scmr (secondary metabolite regulator) for the production of secondary metabolites in the same bacterium. Removal of this regulator then led to overproduction of some secondary metabolites by more than 200-fold ^82^.

However, there is another simple process that can be applied to the screening of microbes for epigenetic-induced amplification of genetic clusters and/or modified metabolites. This is to simply grow two or more microbes in the presence of each other. This is often thought to be a relatively new concept but in fact was being investigated in the pharmaceutical industry in the 1970s using a device known as the EcoLogen, which was effectively four vessels arranged around a central chamber that could be individually closed with solid gates or selective filters. One microbe in the center could be influenced by chemical entities, well before any knowledge of quorum sensors, as the operator desired. There are only three academic references to the use of this device ^83– 85^, but the author used it quite extensively in industry in the late 1970s. However, results were difficult to interpret at times because of the lack of sensitive analytical systems 30-plus years ago.

However, today, there are multiple reports of novel agents coming from mixed culture techniques, with perhaps the first one showing a novel bioactive product being the report from the Fenical group at the Scripps Oceanographic Institution in 2001 of pestalone. This metabolite arose from challenging the marine-derived fungus *Pestalotia* spp. with a marine α-proteobacterium ^86^, which was followed 4 years later by the report of the marine-derived fungus *Libertella* spp. yielding new cytotoxic diterpenoids, libertellenones A–D, when co-fermented with the same α-proteobacterium ^87^. Later work from the same group, but using the marine-sourced fungus *Emericella* spp. and culturing with the obligate marine bacterium *Salinispora arenicola*, produced the cyclic depsipeptides known as emericellamides A and B ^88^.

In 2014, the Wolfender group published an excellent paper on the methodology for studying microbial interactions in 12-well plates. This mimicked, to some extent, methods used many years ago in industry for the production of novel antibiotics, where one microbe was grown on an agar slant then a liquid culture of another microbe or supplemented medium was added and the presence of induced activity was checked against suitable test microbes. The Wolfender group used modern analytical techniques to demonstrate the production of novel metabolites from this process ^89^. A very interesting variation on the same theme is the one recently published by Barkal *et al.*, where microfluidic techniques are used followed by untargeted metabolomics ^90^.

Thus, one might well state that old techniques often used in the 1960s and 1970s when pharmaceutical houses were the major sources of natural product-derived drug candidates have now met up with modern analytical systems. Unfortunately, except for an occasional patent or publication well after the programs had been shut down, only the memories of the scientists involved are left, as publication was not encouraged and, frequently at that time, the methodologies were proprietary.

## Conclusion

### Is there hope for interesting molecules to come from these multiplex types of screening systems?

From the examples that have been given above, the interplay of very talented academic scientists with state-of-the-art analytical systems bodes well for finding novel agents from (mainly) microbial sources, even though frequently the nominal starting source is not a microbe. As shown in the case of the sponge metabolites from *Theonella swinhoei* Y, the complex molecules produced have significant activity and their structures are ones that no synthetic chemist would ever conceive in the absence of a similar compound. In addition, until that work was reported, the investigation of as-yet-uncultivated microbes from a metabolome aspect was a problem few scientists would even “touch”. Currently, that specific paper ^9^ from 2014 has over 150 citations in the Scopus database at the time of writing. In addition, the recent paper from the Piel group should also be required reading for scientists interested in the potential of this type of technology ^11^.

What is also of import is the realization that in addition to the discovery that significant numbers of marine-sourced agents have a microbe in their background, important bioactive plant-derived compounds such as the taxanes, camptothecins, and vinca alkaloids have endophytic microbes in their “background”, and in the case of maytansine, there is no doubt that the molecule is bacterial in origin ^91^.

The “systems integration” demonstrated above also bodes well for investigations of the enormous potential of both terrestrial and marine microbes and their associated “hosts”. The oceans cover 70% of the Earth’s surface and numbers of microbes per cm ^3^ of seawater alone run between 10 ^3^ and 10 ^5^. It should be remembered that a suspension of 10 ^5^ *E. coli* per mL (1 cm ^3^) is a clear solution to the human eye. When one also considers that ~50% wet weight of a sponge is composed of single-celled organisms (not all eubacteria or fungi), the numbers of potential sources are incalculable if one also includes the microbial content of the seabed. Some relevant recent examples will give an idea of the potential.

In the marine area, the Fenical group and collaborators at the Scripps Oceanographic Institution in San Diego have published extensively on the potential of marine microbes, usually free-living but at times associated with invertebrates. The examples given earlier on products from co-culture are one aspect; in addition, papers from long-time collaborators Jensen and Moore, and later Dorrestein, give further insight into the vast areas that still have to be investigated ^24, 25, 92– 94^. These investigations, when coupled to the methodologies reported by the Piel group on marine-sourced but as-yet-uncultivated microbes (see earlier section), demonstrate the potential of these sources to uncover novel agents that may result from using the “modernized grind and find” and coupling to the latest phenotypic screening techniques.

Finally, one could even postulate that “all that is old is new again” in this field, as older concepts and some early reports of potential novel agents in strange places have now met up with the necessary tools to investigate these areas.

## Abbreviations

HPLC, high-performance liquid chromatography (early versions had “pressure” instead of “performance”); IMS, imaging mass spectroscopy; LLE, lead-like enhanced; MS ^n^; mass spectroscopy (the superscript is a number and means that various further MS fragmentation patterns can be analysed); NMR, nuclear magnetic resonance.
